# Genome-wide molecular evolution analysis of the *GRF* and *GIF* gene families in Plantae (Archaeplastida)

**DOI:** 10.1186/s12864-024-10006-w

**Published:** 2024-01-18

**Authors:** Xinghao Chen, Jun Zhang, Shijie Wang, Hongyu Cai, Minsheng Yang, Yan Dong

**Affiliations:** 1 Forest Department, Forestry College, Hebei Agricultural University, Baoding, China; 2 Hebei Key Laboratory for Tree Genetic Resources and Forest Protection, 071000 Baoding, People’s Republic of China

**Keywords:** *GRF* gene family, *GIF* gene family, Evolution, Protein–protein interaction, Expression analysis

## Abstract

**Background:**

Plant growth-regulating factors (GRFs) and GRF-interacting factors (GIFs) interact with each other and collectively have important regulatory roles in plant growth, development, and stress responses. Therefore, it is of great significance to explore the systematic evolution of *GRF* and *GIF* gene families. However, our knowledge and understanding of the role of *GRF* and *GIF* genes during plant evolution has been fragmentary.

**Results:**

In this study, a large number of genomic and transcriptomic datasets of algae, mosses, ferns, gymnosperms and angiosperms were used to systematically analyze the evolution of *GRF* and *GIF* genes during the evolution of plants. The results showed that *GRF* gene first appeared in the charophyte *Klebsormidium nitens*, whereas the *GIF* genes originated relatively early, and these two gene families were mainly expanded by segmental duplication events after plant terrestrialization. During the process of evolution, the protein sequences and functions of *GRF* and *GIF* family genes are relatively conservative. As cooperative partner, *GRF* and *GIF* genes contain the similar types of cis-acting elements in their promoter regions, which enables them to have similar transcriptional response patterns, and both show higher levels of expression in reproductive organs and tissues and organs with strong capacity for cell division. Based on protein–protein interaction analysis and verification, we found that the GRF–GIF protein partnership began to be established in pteridophytes and is highly conserved across different terrestrial plants.

**Conclusions:**

These results provide a foundation for further exploration of the molecular evolution and biological functions of *GRF* and *GIF* genes.

**Supplementary Information:**

The online version contains supplementary material available at 10.1186/s12864-024-10006-w.

## Background

There is increasing evidence that large numbers of transcription factors have important roles in the regulation of plant growth and development [1]. Growth-regulating factors (GRFs) are plant-specific transcription factors that regulate plant growth, development, and stress responses [2–4]. Since the discovery of *GRF* genes in rice in 2000 [5], their structures and functions have been extensively studied. Subsequently, GRF-interacting factors (GIFs) that interact with GRFs were also identified in *Arabidopsis thaliana* [6]. There is considerable interest in the GRF–GIF complex as a functional unit that has important roles in various aspects of plant growth and development [7, 8].

The N-termini of GRF proteins contain highly conserved QLQ and WRC domains [9]. The QLQ domain consists of the highly conserved Gln-Leu-Gln (QX_3_LX_2_Q) motif and its adjacent residues [5]; it performs transcriptional activation functions by interacting with the SNH domain in GIF proteins [10]. The WRC domain consists of three cysteines and one histidine residue (CX_9_CX_10_CX_2_H, C_3_H motif) as a DNA-binding domain, which can regulate the expression of downstream target genes via binding to *cis*-acting elements [11]. In contrast to the N-termini, the C-termini of GRF proteins considerably vary in length and amino acid residue composition, exhibit only low-to-moderate sequence similarity [7], and contain multiple shorter amino acid motifs (e.g., TQL [Thr, Gln, Leu], GGPL [Gly, Gly, Pro, Leu], and FFD [Phe, Phe, Asp] motifs) [12–14]. Numerous studies have shown that *GRF* genes play important roles in leaf growth [15], flower organ development [16, 17], grain size [18], root growth [3], and regulation of plant organ lifespan [2]. Additionally, *GRF* genes can act as defense signals and in stress responses by coordinating plant growth, such as increased resistance to drought and salt stress in *A. thaliana* overexpressing the *AtGRF7* gene [4]; the downstream target genes of *AtGRF1* and *AtGRF3* are mostly involved in defense responses and disease resistance processes [19].

GIF proteins are a class of transcriptional co-activators in plants, which are functionally homologous to human SYT transcriptional co-activators and belong to the SSXT superfamily [20]. The N-termini of GIF proteins contain a highly conserved SNH domain that can directly interact with the QLQ domain of GRF proteins [7]. The C-terminal region has transactivation activity and is rich in Gln (Q) and Gly (G), and thus, the C-terminal of GIF proteins is called the QG domain [6]. GIF proteins also have important biological functions. The overexpression of *GIF* genes can promote organ growth and enhance the activities of GRF proteins [2, 6, 20–23]. *GIF2* and *GIF3* genes in *A. thaliana* can promote cell proliferation and affect leaf size [24]. Additionally, *GIF* genes play important roles in internode growth [23], lateral root development [25], and response to heavy metal stress [26].

The partnership between GRF and GIF proteins has been demonstrated in multiple species; nearly all GRF and GIF proteins in *A. thaliana* can interact with each other [17, 27, 28]. Additionally, the functions of GRF–GIF fusion proteins have been extensively studied. In wheat, a GRF–GIF chimeric protein promotes plant regeneration, improves transformation efficiency, and facilitates the application of gene editing [29]. A GRF–GIF fusion protein can increase chlorophyll content and delay leaf senescence in *A. thaliana* [30]. The overexpression of *ZmGRF11-ZmGIF2* and *ZmGRF2-ZmGIF3* genes resulted in delayed bolting but accelerated inflorescence stem growth, compared with wild-type *A. thaliana* [13]. Overall, GRF and GIF, along with their transcription complexes, are essential regulatory proteins during plant growth and development.

Considering the critical roles of *GRF* and *GIF* genes in diverse biological processes, there has been extensive research concerning their gene families and functions. For example, 9 *GRF* genes and 3 *GIF* genes were found in *A. thaliana* [12, 24]; 11 *GRF* genes and 3 *GIF* genes were found in rice [31]; 13 *GRF* genes and 8 *GIF* genes were identified in wheat [32]; and 14 *GRF* genes and 3 *GIF* genes were found in maize [13]. However, there have been no studies regarding analyses of the expansion, evolution, interaction, and tissue-specific expression of these two gene families in aquatic and terrestrial plants. Therefore, to further characterize the evolutionary histories of the *GRF* and *GIF* gene families, we performed genome-wide identification of GRF and GIF proteins in 29 species ranging from aquatic algae to angiosperms. We sought to gain a more comprehensive understanding of the origin, taxonomy, structural characteristics, and phylogenetic relationships of GRF and GIF proteins, as well as a preliminary understanding of their molecular evolutionary mechanisms during the process of evolution; the findings are expected to provide a basis for further exploration of their related biological functions.

## Results

### Genome-wide identification of GRF and GIF family genes

To better elucidate the origin, expansion, and evolutionary histories of the *GRF* and *GIF* gene families, we first explored the genomes of 26 green plants using HMMER and BLASTP, including chlorophytes (*Chlamydomonas reinhardtii*, *Chlorella variabilis* NC64A, *Coccomyxa subellipsoidea* C-169, *Micromonas pusilla* CCMP1545, *Ostreococcus tauri*, and *Monoraphidium neglectum*), charophytes (*Klebsormidium nitens* NIES-2285 and *Chara braunii*), bryophytes (*Marchantia polymorpha*, *Physcomitrium patens*, and *Sphagnum fallax*), pteridophytes (*Selaginella tamariscina* and *Salvinia cucullata*), gymnosperms (*Ginkgo biloba*, *Picea abies*, and *Gnetum montanum*), and angiosperms (*Amborella trichopoda*, *A. thaliana*, *Glycine max*, *Nicotiana tabacum*, *Populus trichocarpa*, *Vitis vinifera*, *Zea mays*, *Oryza sativa*, *Asparagus officinalis*, and *Musa balbisiana*). To identify *GRF* and *GIF* genes in species that have diverged earlier in evolutionary history, we also explored the genomes of rhodophytes (*Chondrus crispus* and *Cyanidioschyzon merolae*) and glaucophytes (*Cyanophora paradoxa*).

**Fig. 1:**
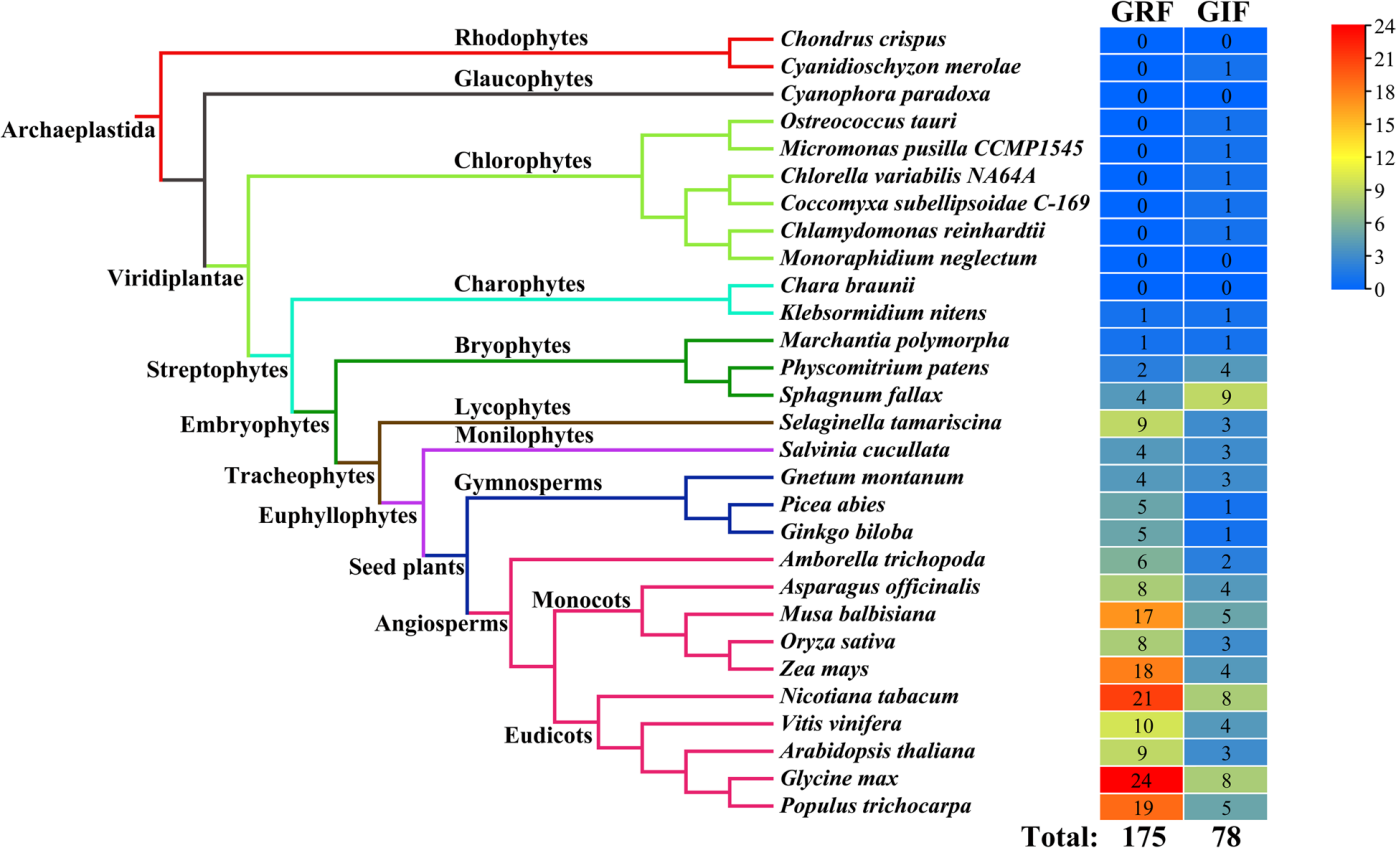
Numbers of *GRF* and *GIF* genes identified in 29 species. The total number of GRF and GIF proteins identified in each plant genome is indicated on the right. The species tree is constructed using OrthoFinder software [33]

Redundant sequences were removed, and the candidate GRF and GIF protein sequences were examined to confirm whether they contained the corresponding domains. Specifically, GRF proteins were required to contain QLQ and WRC domains, whereas GIF proteins were required to contain SSXT domains. After screening and sorting, we finally identified 175 *GRF* and 78 *GIF* genes from the 29 genomes described above (Fig. 1). *GRF* genes first appeared in the charophyte *K. nitens*, whereas *GIF* genes had already appeared in the rhodophyte *C. merolae*. However, compare to the *GIF* gene family, the number of members of the *GRF* gene family considerably increased with evolution, particularly in angiosperms. The analysis of gene gain and loss also confirmed that there were 101 *GRF* gene gain events and 96 *GRF* gene loss events, whereas *GIF* gene loss was greater than gene gain in 29 species (Fig. S1). These findings indicated that *GRF* genes have a later origin than *GIF* genes, although *GRF* genes have expanded faster during the evolution of plants.

### Phylogenetic and protein motif analysis of GRF and GIF family genes

**Fig. 2:**
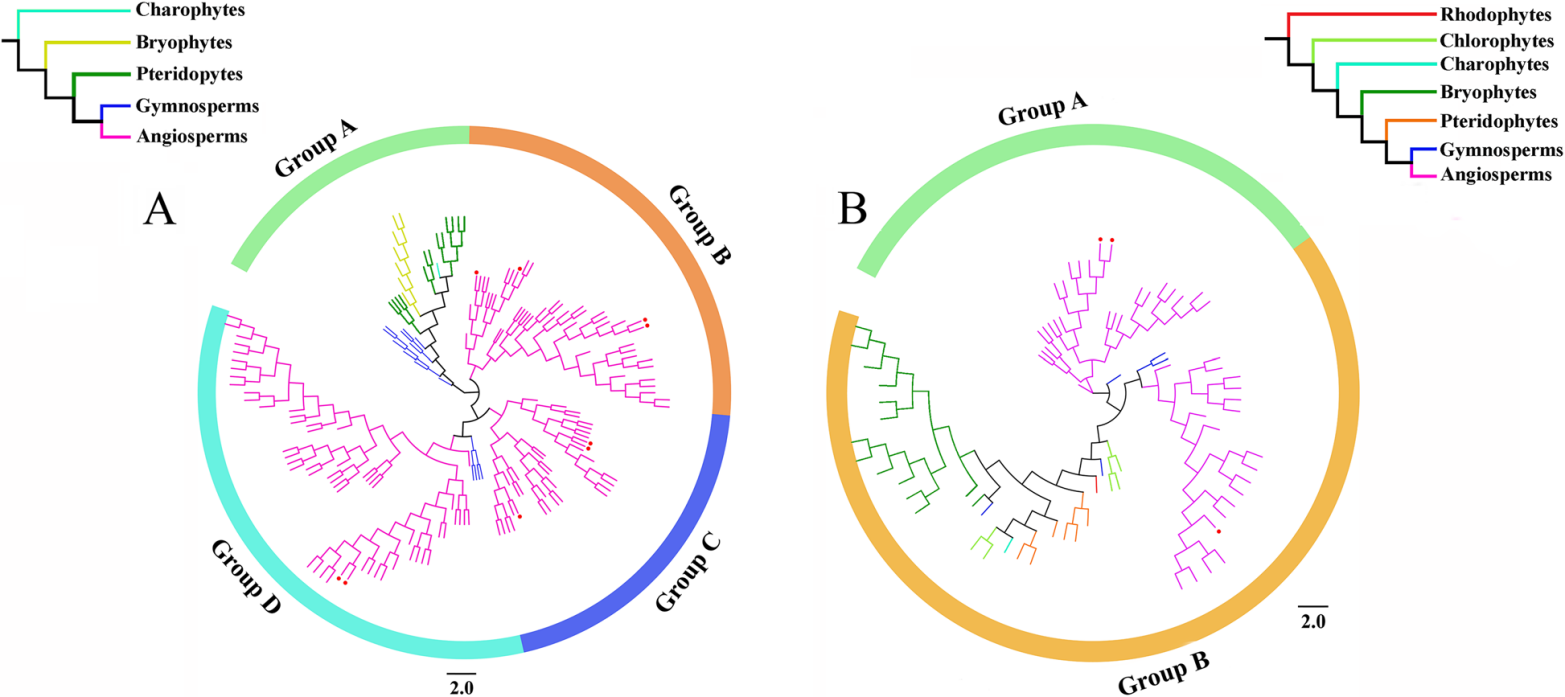
Phylogenetic trees of *GRF* (**A**) and *GIF* (**B**) family genes constructed by the maximum likelihood method. Simplified trees with colored branches used to distinguish species from different plant lineages. The branches of the *GRF* and *GIF* genes in *A. thaliana* are marked with red dots

To explore the evolutionary relationships of genes in different species, phylogenetic trees of *GRF* and *GIF* genes were constructed by maximum likelihood and Bayesian methods. The two methods generally produced consistent topologies, indicating a high degree of accuracy in the constructed phylogenetic trees of *GRF* and *GIF* genes (Fig. 2, S2-S5). To facilitate subsequent analyses, we used the phylogenetic trees constructed by maximum likelihood method. According to the topological structure, the *GRF* genes were divided into four groups. Groups B and C contained only angiosperm *GRF* genes, and Group D contained the largest number of *GRF* genes (*n* = 61). With the exception of a few genes from gymnosperms, most genes in Group D were also from angiosperms. Group A contained the fewest genes (*n* = 31) and did not include angiosperm *GRF* genes, indicating that Group A represents the earliest evolutionary branch (Fig. 2A).

Phylogenetic analysis showed that *GIF* family genes could be divided into two groups. Group A included 26 *GIF* genes, all of which were from angiosperms, including *Arabidopsis AtGIF2* (NP_563619.1) and *AtGIF3* (NP_567194.1); Group B contained a large number of *GIF* genes from species covering most of the plant lineages used in this study (*n* = 52), including *Arabidopsis AtGIF1* (NP_198216.2) (Fig. 2B).

**Fig. 3:**
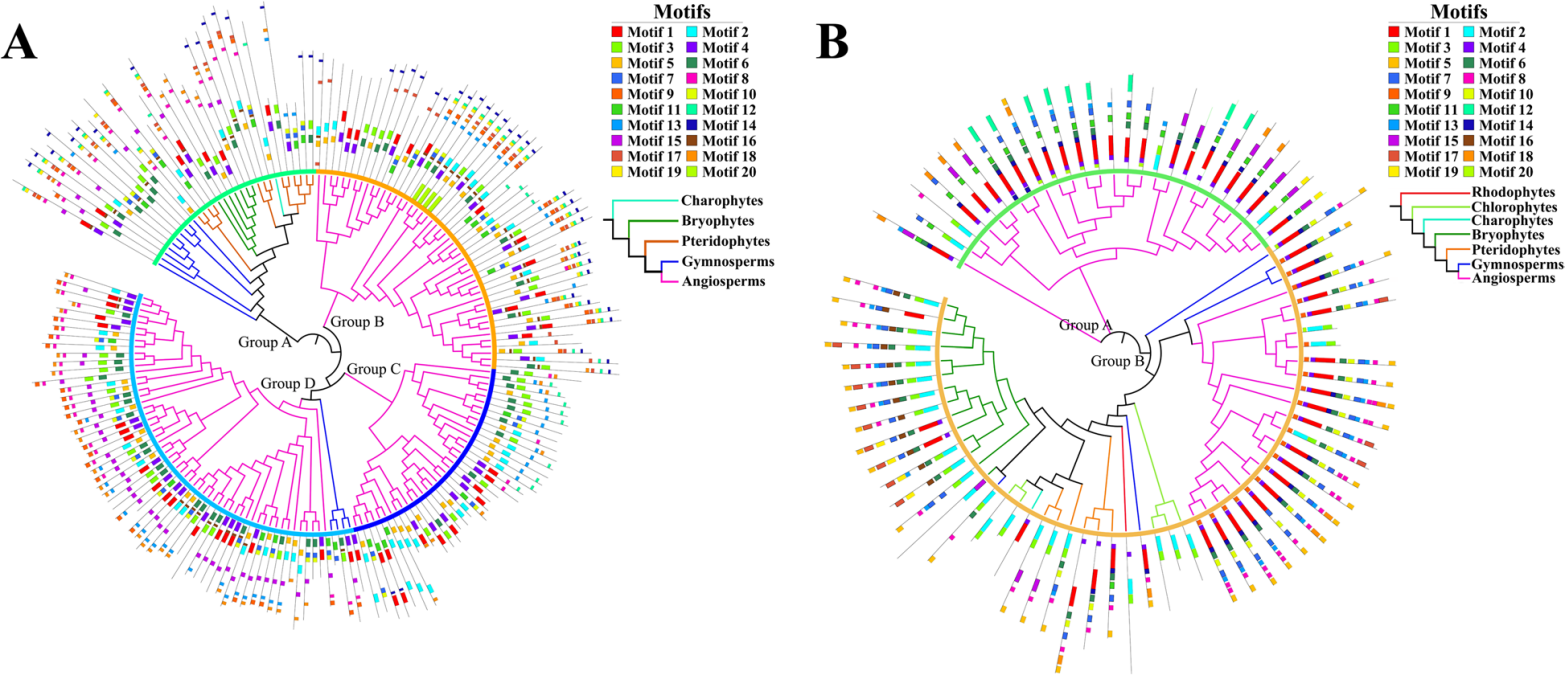
Analysis of conserved motif features in GRF (**A**) proteins and GIF (**B**) proteins. Colors used for the tree branches are the same as in Fig. 2. The outermost circle represents the *GRFs* or *GIFs* motif

**Fig. 4:**
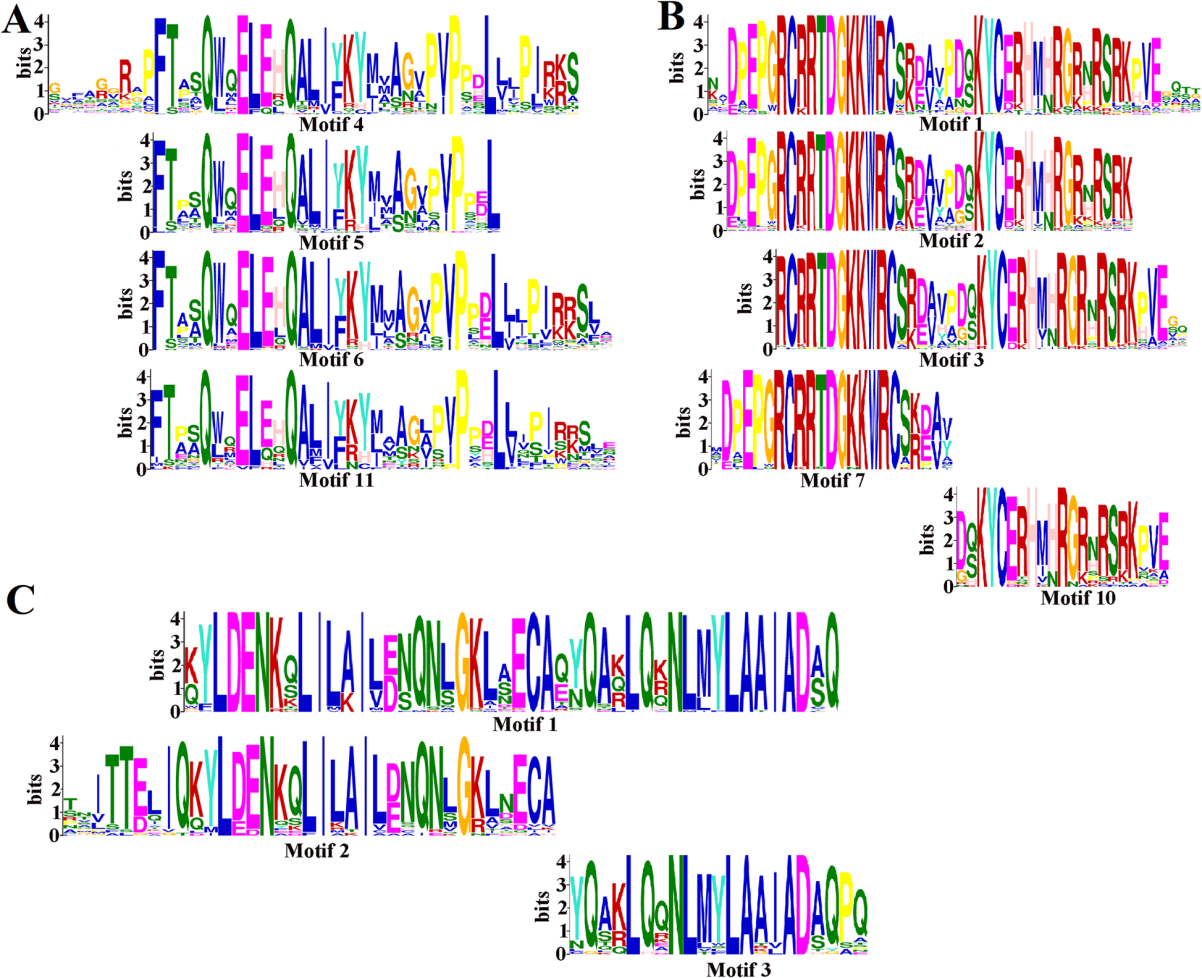
Motif feature analysis of QLQ (**A**) and WRC domains (**B**) in GRF proteins and the SSXT domain (**C**) in GIF proteins

Protein motifs are short conserved sequences that are common to a group of related proteins and generally have biological functions. Identification and analysis of protein motifs is crucial for understanding the function and mechanism of proteins. The N-termini of GRF protein sequences in the same group have motifs that contain or comprise the complete QLQ and WRC domains, but the types of motifs are not identical. For example, motifs 4, 5, 6, and 11 all contain the complete QLQ domain; motifs 1, 2, and 3 also contain the complete WRC domain; and motifs 7 and 10 together comprise the WRC domain (Fig. 3A, Fig. 4A-B, Table S1). The C-termini of GRF protein sequences in the same group are generally highly conserved, but the C-terminal motifs are diverse because different types of amino acid residues are present in the C-termini of GRF proteins in different groups. GRF proteins in both Groups A and B contain more amino acid residues in their C-termini, and the types of amino acids are similar. Group A contains more instances of motif 8, whereas Group B contains more instances of motif 13. Both motifs correspond to FFD motifs, but motif 8 is more highly conserved. Groups C and D contain fewer amino acid residues. Group C contains more FFD motifs (motif 13) and GGPL motifs (motif 12), whereas Group D contains more FFD motifs (motifs 8 and 13) and TQL motifs (motifs 9 and 18) (Fig. S6). In summary, GRF proteins have conserved N-termini but diverse C-terminal amino acid sequences.

GIF proteins are highly conserved. The SSXT domain of seed plants is completely contained within motif 1, whereas motifs 2 and 3 together constitute the conserved SSXT domain in ferns, mosses, and algae. The C-termini of GIF proteins are also diverse; the C-termini in Group A mainly contain motifs 12 and 18, whereas motifs 5 and 17 are dominant in Group B (Fig. 3B, Fig. 4C, Table S2).

### Analysis of cis-acting elements in the promoter regions of *GRF* and *GIF* genes

**Fig. 5:**
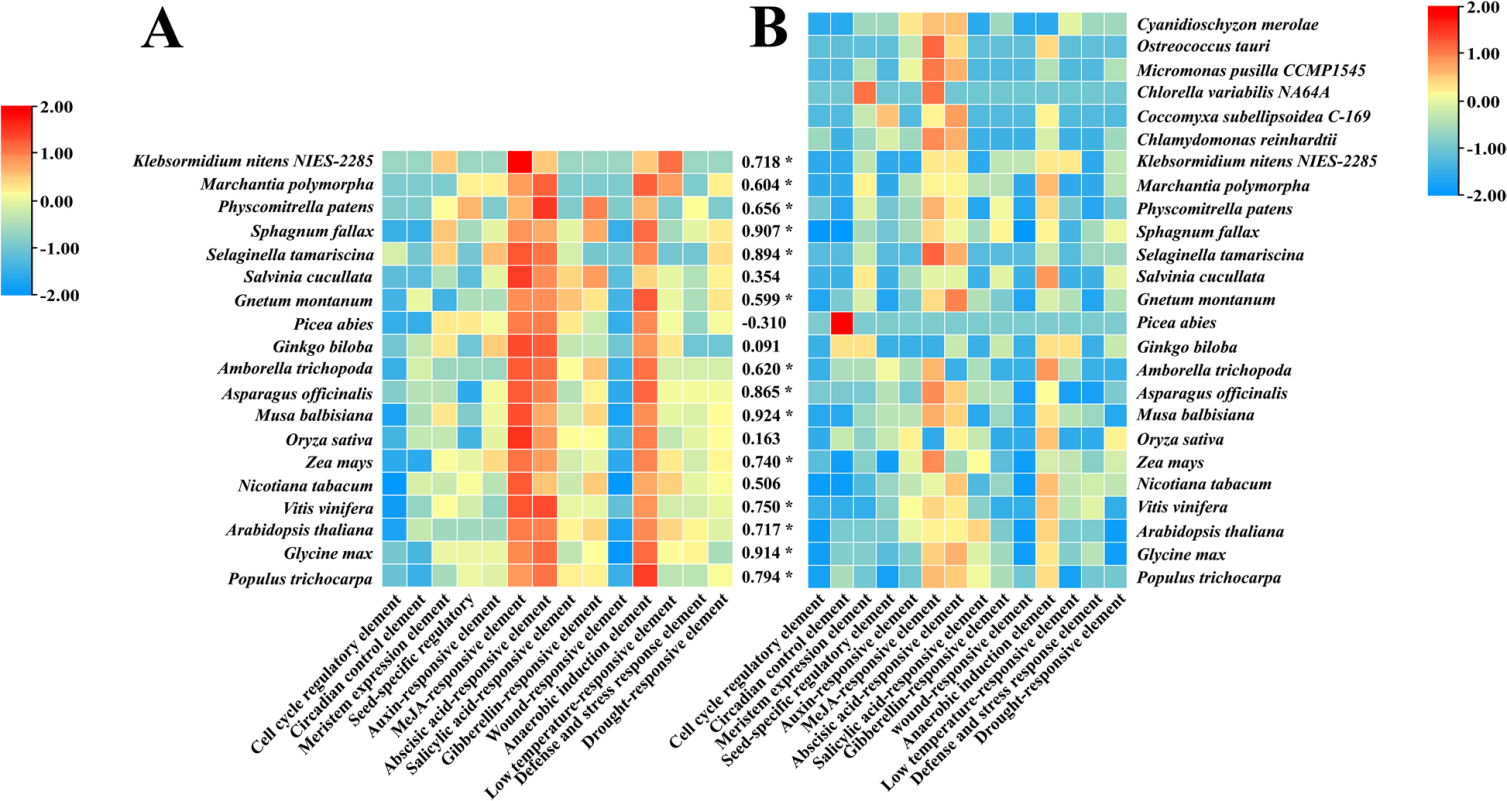
Analysis of the types, numbers, and correlations of *cis*-acting elements in the promoter regions of *GRF* (**A**) and *GIF* (**B**) gene families in different species. The species are shown on the left or right and the *cis*-acting elements are indicated at the bottom of each column. * Indicates that the difference is significant (*P < 0.05*)

The *cis*-acting elements are the DNA sequences present in the promoter region of the gene, which are involved in the regulation of gene expression by binding to transcription factors [34]. In addition to the core elements (TATA-box, CAAT-box and CCAAT-box), 15 *cis*-acting elements related to light response, growth and development, hormone response, and various stress-responsive elements were detected in the promoter regions of 175 *GRF* and 78 *GIF* genes (Table S3–4). Light-responsive elements were the most abundant in the promoter regions of each *GRF* and *GIF* gene, indicating that they may play an important role in mediating the regulation of the light signaling components of the *GRF* and *GIF* genes. *GRF* and *GIF* genes in different species all contained same types of *cis*-acting elements, with no significant differences in the number of *cis*-acting elements, indicating that the functions of *GRF* and *GIF* genes are highly evolutionarily conserved (Fig. S7–8). The number of most cis-acting elements in the promoter regions of *GIF* genes in *P. abies* was relatively small, but during the process of evolution, the number of *cis*-acting elements in *GRF* family genes in most species has been significantly positively correlated with the number of *cis*-acting elements in *GIF* family genes. With the exception of light-responsive elements, the MeJA-responsive and abscisic acid-responsive elements (i.e., hormone response elements) and the anaerobic induction response elements (i.e., stress-responsive elements) are widely distributed in the *GRF* and *GIF* gene families in all species, suggesting that *GRF* and *GIF* genes coevolved as partners and have similar response patterns in terms of transcription and protein expression (Fig. 5).

### Evolutionary analysis of *GRF* and *GIF* genes

To elucidate the evolutionary basis of the functional diversification of GRFs and GIFs in each group, we analyzed the genetic difference based on the nonsynonymous-to-synonymous rates ratio. In the *GRF* gene family, the genetic distance is smallest between Groups A and B (0.411), indicating that the *GRF* gene sequences have high similarity between these two groups (Table 1). The genetic distance is largest between Groups B and C (0.542), indicating that the sequences have low similarity between these two groups. The genetic distance between the two groups in the *GIF* gene family is only 0.389, which is smaller than all comparisons in the *GRF* gene family; thus, the *GIF* family genes are more highly conserved than the *GRF* family genes (Table S5).

**Table 1:** Genetic distances between different groups of *GRF* family genes

GRF	Group A	Group B	Group C	Group D
Group A		0.055	0.069	0.060
Group B	0.411		0.078	0.068
Group C	0.507	0.542		0.074
Group D	0.415	0.425	0.508	

Data in lower triangles represent genetic distances between different groups, whereas data in upper triangles represent corresponding standard errors

To clarify the evolutionary basis of *GRF* and *GIF* family genes, the ratios of non-synonymous to synonymous substitutions (ω = *K*a/*K*s) in each group were calculated. In all *GRF* and *GIF* gene family groups, the mean number of non-synonymous sites was much higher than the mean number of synonymous sites (SS), and the mean ω values were all considerably less than 1, indicating strong purifying selection during evolution. Additionally, the mean ω value of each group in the *GIF* gene family was lower than the mean ω value of each group in the *GRF* gene family, indicating slower evolution of *GIF* family genes; this strengthened the conclusion that *GIF* family genes are more highly conserved (Table 2).

**Table 2:** Molecular evolution of *GRF* and *GIF* gene family genes

Gene family	Group	NSS	SS	Ka	Ks	Ka/Ks
GIF	A	223.51	61.49	0.2022	0.6870	0.294
	B	120.42	35.58	0.1562	0.7552	0.207
GRF	A	229.28	73.72	0.2830	0.7288	0.388
	B	237.93	74.07	0.2636	0.6587	0.400
	C	161.84	48.16	0.2445	0.6380	0.383
	D	200.52	60.48	0.1480	0.6883	0.215

### Synteny analysis of *GRF* and *GIF* genes

**Fig. 6:**
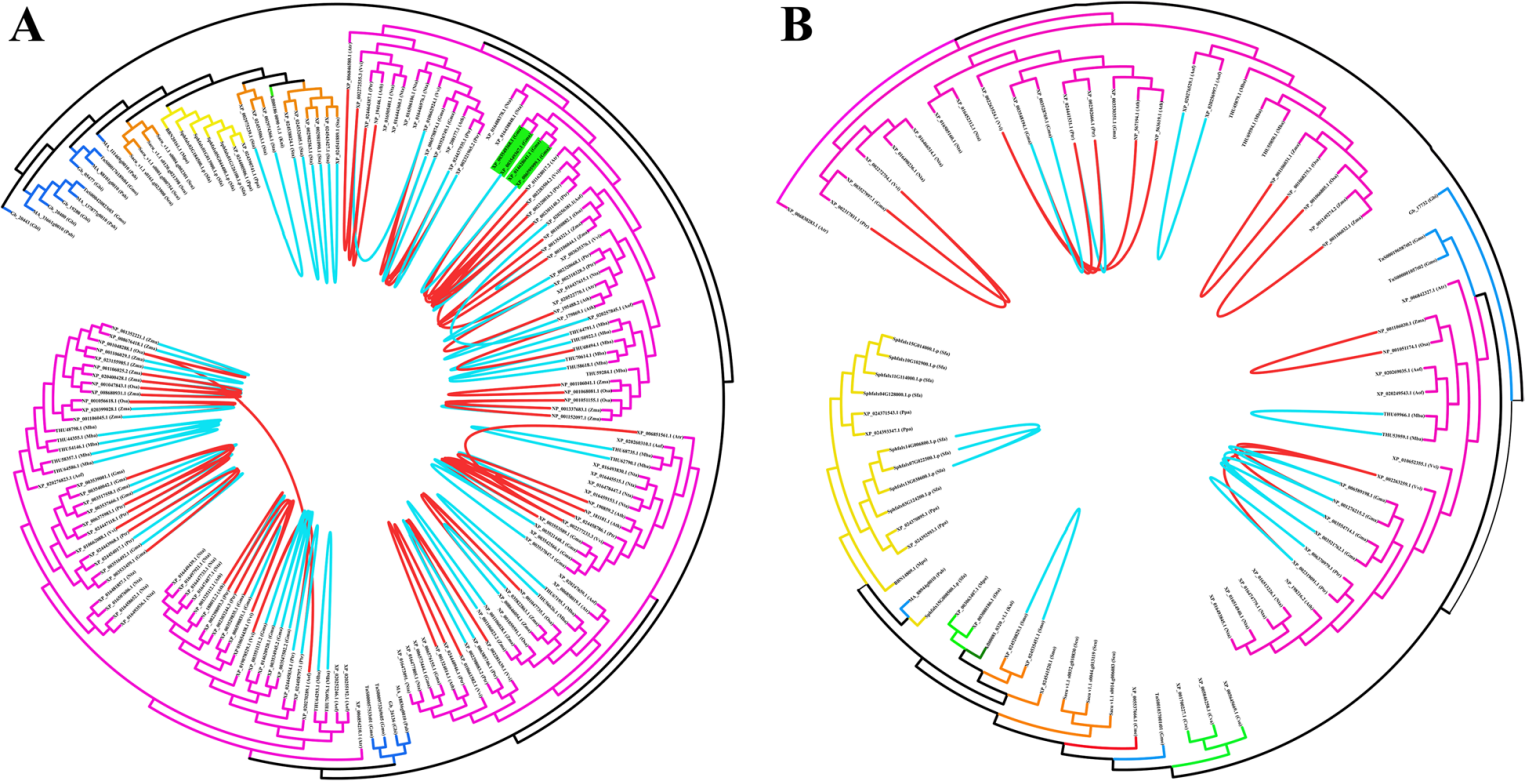
Results of synteny analysis of *GRF* (**A**) and *GIF* (**B**) family genes. Blue lines represent intragenomic gene duplication events, whereas red lines represent intergenomic gene duplication events. The gene IDs with green shading are two tandem duplication events in the *GRF* family genes

Gene duplication is a major force for the generation of gene families, which can not only lead to the functional differentiation of duplicate genes, but also promote the evolution of genomes and species.. To understand the gene duplications and evolutionary histories of *GRF* and *GIF* genes, gene duplication events in the genomes of 29 species were analyzed, including intra- and intergenomic segmental duplication events, as well as tandem replication events. Intragenomic segmental duplication events of *GRF* and *GIF* genes were detected in bryophytes, ferns, and angiosperms, whereas intergenomic segmental duplication events were only found in angiosperms. In *GRF* family genes, only two tandem replicating gene pairs were detected in the angiosperm *G. max*, whereas none were detected in *GIF* family genes; thus, tandem replication events presumably made minimal contributions to the expansion of *GRF* and *GIF* gene families. Importantly, no gene duplication events were detected in algae or gymnosperms (Fig. 6). In conclusion, the *GRF* and *GIF* family genes, particularly in angiosperms, were mainly expanded by segmental duplication events.

### Interaction analysis between GRF and GIF proteins

**Fig. 7:**
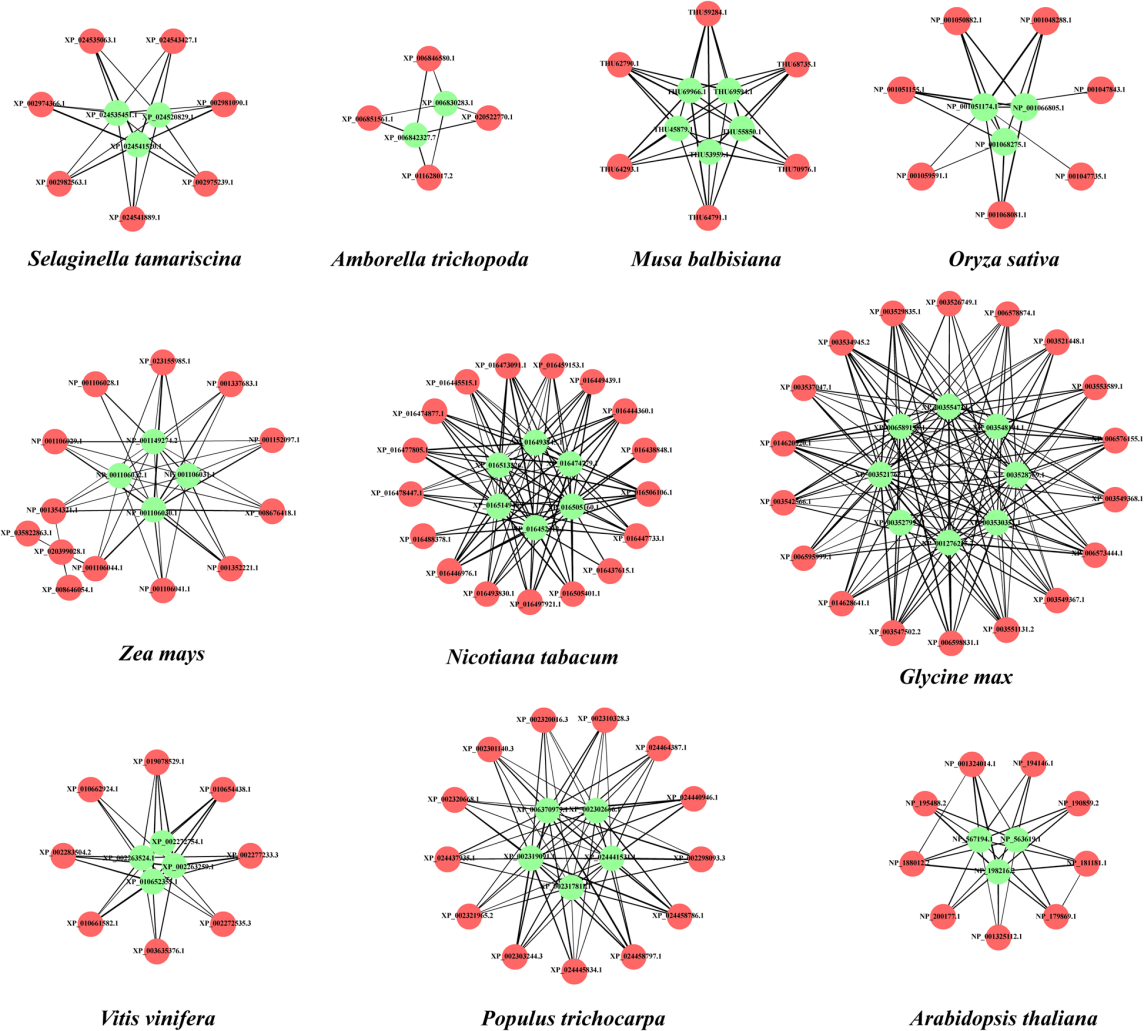
Interaction analysis of GRF and GIF proteins. Red circles represent GRF proteins, whereas green circles represent GIF proteins

The GRF proteins interact with GIF proteins to form a functional transcriptional complex. To explore whether the GRF-GIF protein partnership was conserved during species evolution, the analyses of interactions between GRF and GIF proteins were carried out for the 12 genomes in this study that were included in the STRING database [35] (Table S6). The results showed that GRF and GIF proteins in the charophyte *K. nitens* and bryophyte *P. patens* did not interact with each other. An interaction between a GRF protein and a GIF protein was first found in the pteridophyte *S. tamariscina*; such interactions were extensively observed in angiosperms. In the remaining 10 species, some GRF proteins cannot interact with their GIF proteins; however, with the exception of *N. tabacum*, all GIF proteins can interact with GRF proteins in the same species. Notably, among these interacting GRF and GIF proteins, not all GRF proteins and GIF proteins have a one-to-one interaction. For example, in *O. sativa*, two GRF proteins (NP_001047843.1 and NP_001047735.1) can only interact with one GIF protein (NP_001051174.1); the other four GRF proteins (NP_001048288.1, NP_001050882.1, NP_001051155.1, and NP_001068081.1) can interact with all three GIF proteins (Fig. 7). The results indicated that the majority of GRF proteins can interact with GIF proteins, although they may also have other regulatory modes; this constitutes further evidence that the functions of GIF proteins are generally conserved, whereas the functions of GRF proteins are diverse. Additionally, although the numbers of *GRF* and *GIF* genes have considerably increased during the process of evolution, the mean number of actual interacting GRF–GIF protein pairs constitutes approximately 60% of the number of theoretically possible interaction protein pairs, indicating that there are highly specific interactions between GRF and GIF family proteins (Table S7). This finding supports the conclusion that the functions of GRF and GIF protein families, particularly GIF proteins, have generally been highly conserved during evolution.

### Verification of the interaction between GRF and GIF proteins

To determine whether the GRF-GIF protein partnership was really established in pteridophytes, we used split-ubiquitin yeast two-hybrid system to analyze the protein interaction between a GRF protein and a GIF protein randomly selected from each of charophyte alga *K. nitens*, liverwort *M. polymorpha*, moss *P. patens* and fern *S. tamariscina.*

**Fig. 8:**
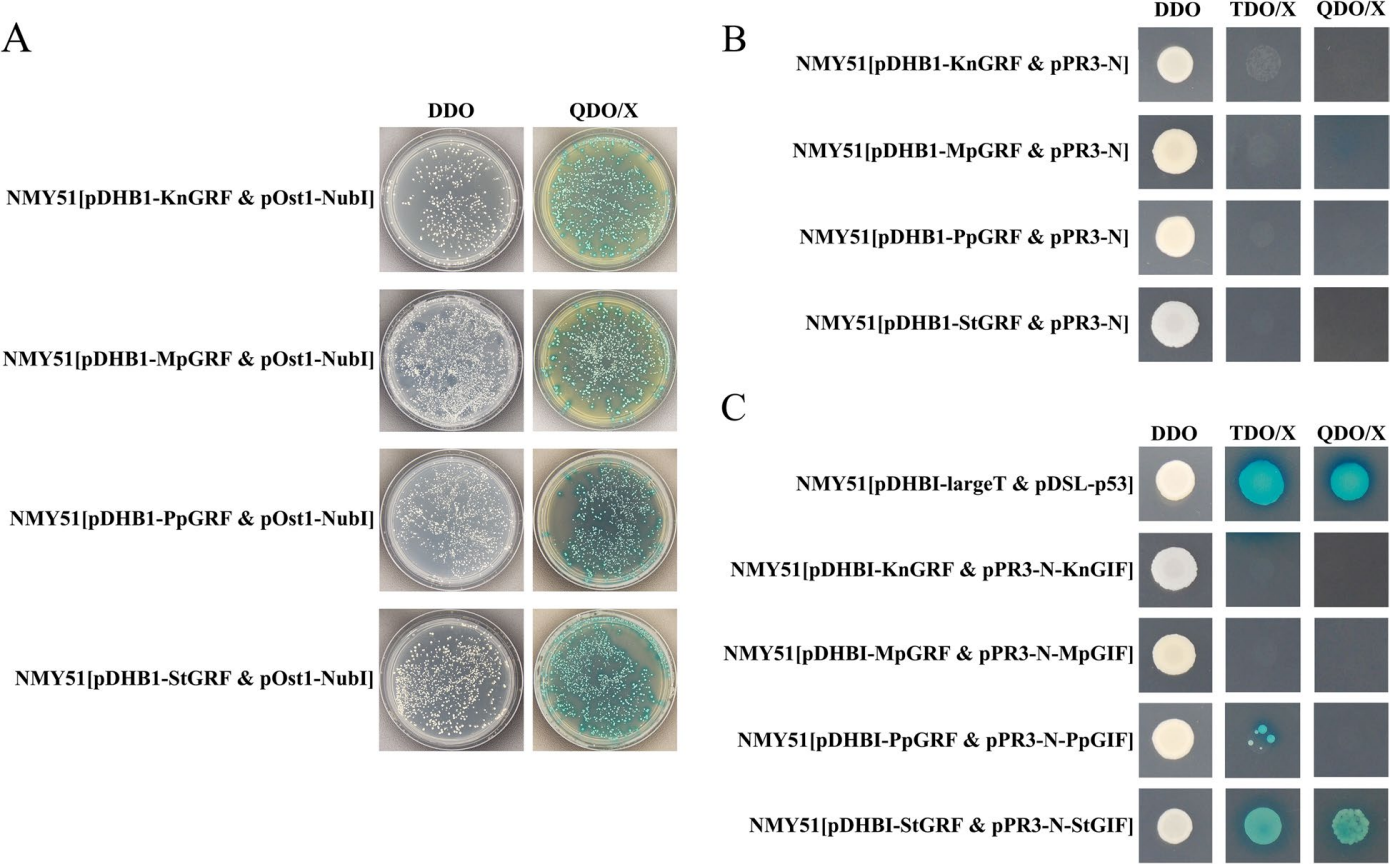
Split-ubiquitin yeast two hybrid assay. **A** Function verification of the bait plasmid pDHB1-GRF; (**B**) Self-activation assay of the bait plasmid pDHB1-GRF; (**C**) Verification of the interaction between GRF and GIF proteins

The *GRF genes* and *GIF genes* from different species were constructed in the bait plasmid pDHB1 and the prey plasmid pPR3-N, respectively. Co-transformation of the bait plasmid pDHB1-GRF and the prey plasmid pOst1-NubI (positive control) in yeast resulted in growth of yeast on all selective media (Fig. 8A), whereas the co-transformation of the bait plasmid pDHB1-GRF and prey plasmid pPR3-N (negative control) did not grow on the TDO/X (SD/−His/−Leu/−Trp/X-a-gal) and QDO/X (SD/−Leu/−Trp/−His/−Ade/X-a-gal) (Fig. 8B), suggesting that all the bait plasmids were functionally well expressed and had no self-activation activity in the split-ubiquitin Y2H system. Among the four species, only the yeast cells co-transformed with pDHB1-StGRF and pPR3-N-StGIF as well as the positive control grew well and turned blue on the TDO/X and QDO/X (Fig. 8C). The results showed that GRF and GIF proteins in pteridophytes could interact with each other, while GRF and GIF proteins in charophytes and bryophytes had no interaction, which was consistent with the results predicted by STRING database.

### Expression analysis of *GRF* and *GIF* genes in different tissues

To analyze the temporal and spatial expression patterns of *GRF* and *GIF* genes, we downloaded gene expression datasets from GEO (Gene Expression Omnibus) and SRA (Sequence Read Archive) databases in NCBI to compared their expression patterns among developmental stages and among different species ranging from aquatic algae to angiosperms that represent terrestrial plant lineages, including *P. patens*, *M. polymorpha*, *S. tamariscina*, *G. biloba*, *P. trichocarpa*, *Z. mays*, *O. sativa*, and *A. thaliana*.

**Fig. 9:**
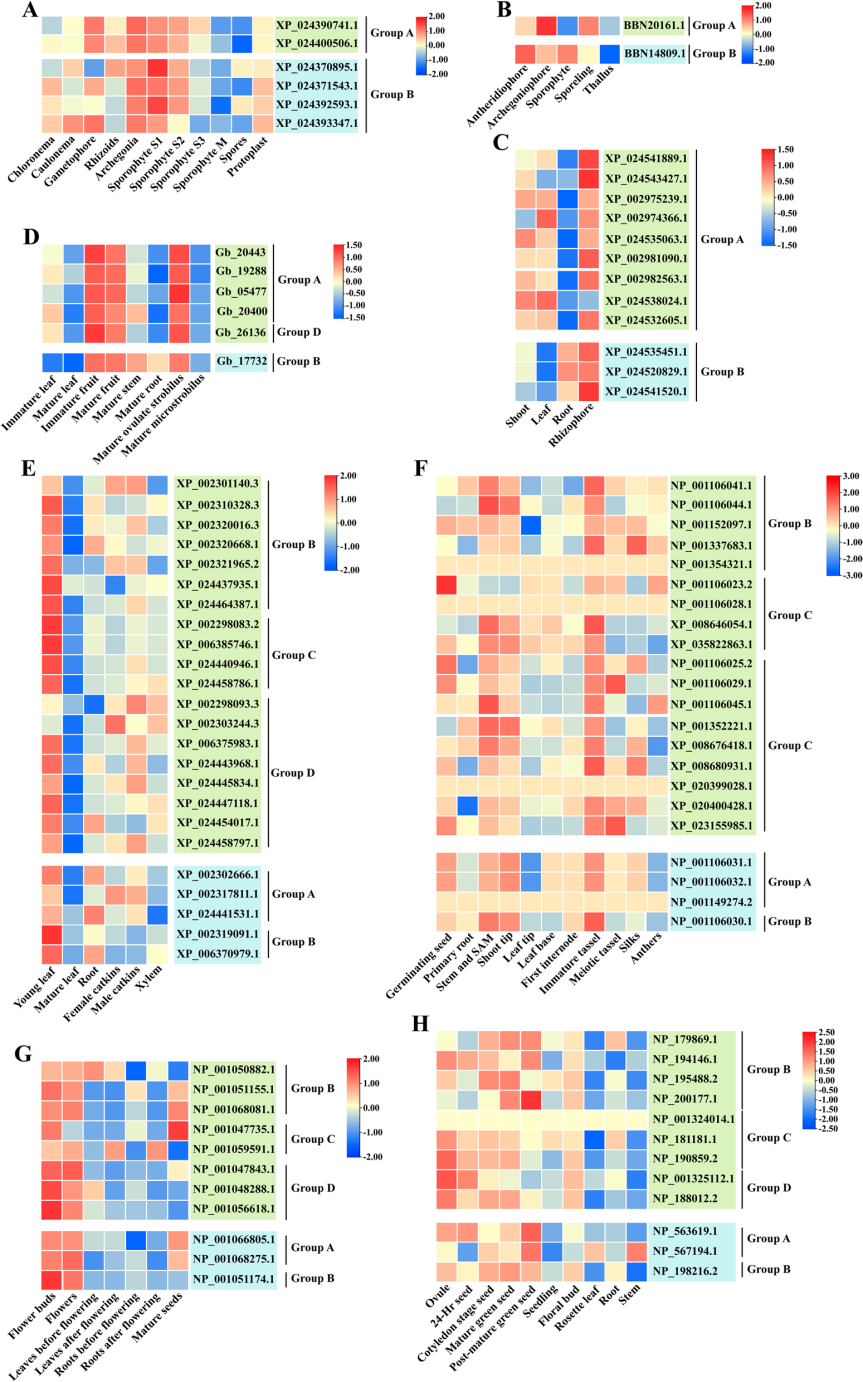
Expression analysis of *GRF* and *GIF* genes in various tissues of different species. The genes are displayed on the right and the tissues used for expression analysis are indicated at the bottom of each column. Red, blue, and yellow indicate high, low, and medium gene expression levels, respectively. The IDs with light green and light blue shading are the *GRF* genes and *GIF* genes, respectively. **A** *Physcomitrella patens*; **B** *Marchantia polymorpha*; **C** *Selaginella tamariscina*; **D** *Ginkgo biloba*; **E** *Populus trichocarpa*; **F** *Zea mays*; **G** *Oryza sativa*; **H** *Arabidopsis thaliana*

In general, the expression patterns of *GRF* and *GIF* genes in different species and tissues were similar, presumably because of the interactions between GRF and GIF proteins. No significant differences were found in the expression patterns of *GRF* or *GIF* genes in similar tissues between different groups. However, *GRF* and *GIF* genes showed opposite expression patterns in the tissues of some species, such as the leaf and root of *S. tamariscina* and the mature root of *G. biloba*. Notably, *GRF* and *GIF* genes generally had higher expression levels in tissues at the growth and development stages, such as young leaves, ovules, and germinating seeds; their expression levels were generally low in mature tissues, such as mature leaves of *G. biloba* and *P. trichocarpa*. Additionally, *GRF* and *GIF* genes were highly expressed in reproductive organs, such as the archegonia and immature sporophytes of *P. patens*, the antheridiophore and archegoniophore tissues of *M. polymorpha*, the female catkins and male catkins of *P. trichocarpa*, and the flowers or floral buds of *O. sativa* or *A. thaliana* (Fig. 9). The above results indicated that *GRF* and *GIF* genes play important roles in the growth and development of plant tissue, as well as the process of reproductive growth.

## Discussion

In this study, two methods were used to identify *GRF* and *GIF* genes in the genomes of 29 species, several of which had been identified in previous studies. The results were consistent with findings from previous studies in plants such as *A. thaliana* [12, 24], *Z. mays* [13]*,* and *O. sativa* [31], indicating the reliability of the results presented here. The number of *GRF* family genes has substantially expanded during evolution, particularly in angiosperms. For example, there are more than 20 *GRF* genes in *N. tabacum* and *G. max*. *GRF* genes were first discovered in the charophyte *K. nitens*, and therefore GRF is a type of streptophyte-specific transcription factor. It has been speculated that *GRF* genes were generated by evolutionary exchange of genes containing the QLQ domain and the WRC domain; they appeared in the ancestors of charophyte plants after the differentiation of green algae into charophytes [10], which is consistent with the present results. Terrestrial plants evolved from semiterrestrial charophytes [36]. The *GRF* gene exists as a single copy, but rapidly expands in terrestrial plants, and plays an important roles in plant growth and development. Therefore, *GRF* genes presumably played important roles during plant terrestrialization. The origin of *GIF* genes is older than the origin of *GRF* genes. In this study, a *GIF* gene was found in the red alga *C. merolae*, suggesting that the gene originated in red algae or from species that diverged earlier. Compared with *GRF* genes, the number of *GIF* genes in all species analyzed was much lower and did not substantially change during species evolution. In summary, *GRF* genes have a later origin than *GIF* genes, but *GRF* genes expanded faster during the evolution of plants.

Both the *GRF* and *GIF* gene families are highly conserved. According to the phylogenetic trees constructed by the maximum likelihood and Bayesian methods, the *GRF* family genes are divided into four groups, but the motifs of the N-termini of GRF proteins in different groups can contain or form complete QLQ and WRC domains; conversely, the C-termini of GRF proteins in different groups contain distinct amino acid motifs, including FFD, GGPL, and TQL motifs, with diverse C-terminal sequences that result in only low-to-moderate sequence similarity [7, 11]. These C-terminal amino acid motifs may have transactivation activity because truncated C-terminal GRF proteins usually lose their transactivation activity in *A. thaliana*, *O. sativa*, and *N. tabacum* [6, 17, 37]. According to the types of motifs, GIF proteins are more highly conserved than GRF proteins, although their C-terminal sequences are also diverse. Additionally, the genetic distances between different groups in the *GRF* and *GIF* gene families are generally small and have been subjected to purification selection during evolution, further indicating conservation of their sequences.

The promoter regions of *GRF* and *GIF* genes contain similar types of *cis*-acting elements, which are mainly related to light response, growth and development, hormone response, and various stress responses; these findings suggest that *GRF* and *GIF* genes have similar response patterns in their transcription and protein expression, which may explain why they can form fusion proteins to perform biological functions in a collective manner. The types of *cis*-acting elements present in the promoter regions of *GRF* and *GIF* genes were similar, and there were no significant differences in number between the different groups; thus, the functions of *GRF* and *GIF* genes have presumably been highly conserved during evolution. For further verification, we explored the functions of nine *GRF* genes in *A. thaliana* (Table 3)*.* Although *GRF* genes in Groups B and C show considerable functional diversity, the *GRF* genes in the three groups play roles in regulating plant growth and development and in responding to stress, consistent with the results of *cis*-acting element analysis; thus, the functions of *GRF* genes may not be specific among different groups, and *GRF* gene functions are presumably conserved. Similarly, three *GIF* genes in *A. thaliana* share functional similarities; all have important roles in regulating plant cell division and plant organ size [6, 20, 24]. In conclusion, *GRF* and *GIF* genes have substantial functional conservation during evolution; because of this cooperative partnership, the presence of similar types of *cis*-acting elements may also explain why GRF and GIF proteins share biological functions.

**Table 3:** Summary of *GRF* gene functions in *A. thaliana*

Group	Name	ID	Biological Function
B	GRF1	NP_179869.1	Controls the embryogenic response [38]. Involved in auxin synthesis, as well as the regulation of circadian rhythm and the cell cycle [39]. Involved in regulating leaf growth, size, and morphology [6, 12]. Involved in root growth [3]. Response to abiotic stress (cold [40] and UV-B radiation [41]) and biotic stress (bacteria [39] and cyst nematode infection [3]. Coordinates interactions between defense signaling and plant growth and development [19, 39].
B	GRF2	NP_195488.2	Regulates the size of organs, such as leaves and cotyledons [6, 12, 42]. Involved in root tissue development [3, 43]. Response to abiotic stress (cold [40] and UV-B radiation [41]).
B	GRF7	NP_200177.1	Involved in chlorophyll synthesis [44]. Response to abiotic stress (cold [40], salinity [45], abscisic acid, and osmotic stress [4]).
B	GRF8	NP_194146.1	Controls the embryogenic response [38]. Involved in chlorophyll synthesis [44]. Related to flower development [16, 46].
C	GRF3	NP_181181.1	Involved in auxin synthesis, as well as the regulation of circadian rhythm and the cell cycle [39]. Controls the growth and development of leaves, as well as the size of organs [2, 6, 12, 42]. Involved in root tissue development [3, 43]. Response to abiotic stress (cold [40] and UV-B radiation [41, 47]) and biotic stress (bacteria [39] and cyst nematode infection [3]). Coordinates the interactions between defense signaling and plant growth and development [19, 39].
C	GRF4	NP_190859.2	Controls the embryogenic response [38]. Regulates leaf, cotyledon, and shoot apical meristem [48]. Regulates circadian rhythm of leaves [49]. Regulates growth under conditions of cold stress [40].
C	GRF9	NP_001324014.1	Controls the embryogenic response [38]. Regulates growth under conditions of cold stress [40]. Regulates leaf growth by controlling cell proliferation in leaf primordia [50].
D	GRF5	NP_188012.2	Stimulates chloroplast division, photosynthesis, and leaf longevity [30]. Related to ovule formation [51]. Regulates leaf cell proliferation, as well as leaf growth and development [2, 52–54]. Regulates growth under conditions of cold stress [40].
D	GRF6	NP_001325112.1	Regulates growth under conditions of cold stress [40]. Plays a positive regulatory role under conditions of nitrogen starvation [55].

The evolution of species and polyploidy events experienced by species in their evolutionary histories favor the expansion of gene families [56–58]. Research has shown that genes encoding interacting proteins tend to be co-retained after whole-genome duplication (WGD) to keep the dosage balance [59]. In the charophyte *K. nitens*, both GRF and GIF only have one gene family member. However, during the evolution of plants, *GRF* genes expanded faster than *GIF* genes through segmental duplication events, which is not in N: N mode and conflicts with the gene dosage balance hypothesis. Similarly, the *CBL* and *CIPK* gene families also exhibit such results during plant evolution. Research suggests that duplicated genes after WGD often have different temporal and spatial expression patterns, which is beneficial for separating two competing genes and keeping the dosage balance in specific tissues [60]. In addition, large DNA segments with synteny relationships in the genome are often traces left after WGD events. Synteny analysis revealed large numbers of intra- and intergenomic segmental duplication events of *GRF* and *GIF* genes, whereas almost no tandem replication events were found, indicating that *GRF* and *GIF* genes were mainly expanded through WGD events.

This study showed that *GRF* and *GIF* genes appeared concurrently in the charophyte *K. nitens*; thus, it is theoretically possible that the interaction between GRF and GIF proteins have been established in this species. Similarly, previous analyses showed that the structures of the *GIF* genes were highly conserved in the evolution of green algae, charophytes, and land plants; therefore, the GRF–GIF protein partnership may also have been established in the ancestral charophyte [10]. However, STRING database analyses showed no interactions between GRF and GIF proteins in the charophyte *K. nitens* and bryophyte *P. patens*, but interactions were present in the pteridophyte *S. tamariscina*. Although GRF and GIF proteins coexist in *K. nitens* and *P. patens*, they may not form a complex for collective activity; however, they evolved into protein partnership to regulate growth and development in pteridophytes. Importantly, all GRF and GIF proteins can interact with each other in *A. thaliana*; this is consistent with the results of previous studies [6, 20, 27], indicating that the results of STRING database analysis have high reliability. In addition, we further verified that the GRF-GIF protein partnership was only established in pteridophytes by split-ubiquitin yeast two-hybrid assay. Among angiosperms, most of the GRF and GIF proteins in each species can interact with each other. Although the *GRF* gene copy number substantially increased during the process of evolution, the number of interacting GRF–GIF protein pairs remained high, indicating highly conserved interactions between GRF and GIF proteins.

Both *GRF* and *GIF* genes have important regulatory roles in many plant tissues and organs [2, 3, 5, 6, 15–18, 20, 23–25]. Similarly, we found that *GRF* and *GIF* genes are highly expressed in the tissues, organs, and developmental stages of most species. Importantly, presumably because of their interaction, *GRF* and *GIF* genes in the same tissues and organs show nearly identical expression patterns. In *A. thaliana*, almost each GRF protein can interact with each GIF protein and both *GRF* and *GIF* genes are highly expressed in developing pistils [16]. *GRF* and *GIF* genes in different species tend to have higher levels of transcription in tissues or organs with strong division ability, such as young leaves and developing seeds. Previous studies have shown that *GRF* and *GIF* genes can promote or maintain cell division [6, 10, 12, 20, 24]; thus, their expression levels are higher in the early growth stages of tissues. However, the decreased expression of *GRF* genes in mature tissues may be caused by *miR396* targeting and induction of the cleavage of *AtGRF* mRNAs [2, 61], which may also be the reason for the opposite expression pattern of *GRF* and *GIF* genes in some tissues, such as leaves and roots of *S. tamariscina* and mature roots of *G. biloba*. Additionally, we found that *GRF* and *GIF* genes are highly expressed in plant reproductive organs; they are indispensable regulators of the development of these organs [10]. In many species, the loss of function of *GRF* or *GIF* genes leads to severe structural and functional defects in floral organs [62], abnormal numbers of stigmas or anthers [17], and reduced fertility [63]. However, there have been few studies of *GIF* genes in aquatic algae. Therefore, further studies are needed to determine the biological functions of *GIF* genes prior to the appearance of *GRF* genes in charophytes, along with their functions in the absence of *GRF* genes.

## Methods

### Identification of *GRF* and *GIF* gene family members

To elucidate the evolutionary histories of the *GRF* and *GIF* gene families, genomic data of 29 plants and algae covering major plant lineages were selected for analysis. The genomic data of 22 species were downloaded from the National Center for Biotechnology Information database (https://www.ncbi.nlm.nih.gov/), *S. fallax* genomic data were obtained from Phytozome v.13 (http://phytozome.jgi.doe.gov) [64], *S. cucullata* genomic data were obtained from downloaded from Fernbase (https://www.fernbase.org/) [65], and *P. abies* genomic data were obtained from PlantGenIE (The Plant Genome Integrative Explorer, http://congenie.org/) [66]. Detailed information concerning the genomic data is presented in Table S8.

First, candidate GRF and GIF protein sequences were explored in the 29 genomes using Hidden Markov Models that corresponded to GRF proteins (PF08879 and PF08880) and GIF proteins (PF05030) downloaded from the Pfam database (http://pfam.xfam.org/) [67] through HMMER3.1 (*P* < 0.001) [68]. BLASTP searches [69] were performed to retrieve GRF protein and GIF protein sequences from the 29 genomes using the identified GRF protein and GIF protein sequences of *A. thaliana* downloaded from the *Arabidopsis* database (https://www.arabidopsis.org/) [70] as respective query sequences. After the candidate sequences obtained by the two methods had been merged and redundant sequences had been manually removed, all GRF protein and GIF protein sequences were submitted to InterProScan [71] and the Conserved Domains Database [72] for verification. GRF proteins were required to contain QLQ and WRC domains, whereas GIF proteins were required to contain SSXT domains.

### Multiple sequence alignment and phylogenetic tree construction

All multiple protein sequence alignments were performed in MAFFT v7.471 with the E-INS-i algorithm [73]. PAL2NAL v.14 was used to convert protein alignments to DNA alignments [74]. The DNA alignments were then trimmed using TrimAl v1.4 [75]. The best-fit substitution model was determined by Modelfinder according to the Bayesian information criterion [76]. IQ-TREE 1.6.8 was used to construct the maximum likelihood phylogenetic tree with 1000 bootstrap replicates [77]. Concurrently, MRBAYES v.3.2.6 on CIPRES (https://www.phylo.org/) was used to construct the Bayesian phylogenetic tree [78]. For Bayesian analysis, two independent Markov chain Monte Carlo algorithms were run simultaneously, with four chains each, for 50,000,000 generations [60]. The final phylogenetic tree was visualized using the online software ITOL v.6 (https://itol.embl.de/) [79]. The species/gene tree reconciliation approach with NOTUNG software to infer gene gains and losses of GRF and GIF [80].

### Analysis of the structure and cis-acting elements of GRF and GIF family genes

MEME online software (http://meme-suite.org/tools/meme) was used to analyze the motifs of GRF and GIF protein sequences, with the following parameters: amino acid length, 6–100; threshold number of motif discovery, 20 [81]. To investigate the *cis*-acting elements, the 1500-bp DNA sequences in the upstream regions of *GRF* and *GIF* genes were analyzed using PlantCARE (http://bioinformatics.psb.ugent.be/webtools/plantcare/html) [82].

### Evolutionary analysis of GRF and GIF family genes

DNASP v6.12.03 software was used to calculate the synonymous substitution rate (*K*a) and non-synonymous substitution rate (*K*s) of aligned CDS sequences, along with the ratio of these rates (*K*a/*K*s) [83]. To calculate the genetic divergence between each group of *GRF* and *GIF* family genes, the Jones-Taylor-Thornton model in MEGA 7.0 software was used to calculate genetic distances based on amino acid sequences [84].

### Synteny analysis of GRF and GIF family genes

To characterize duplication events involving *GRF* and *GIF* genes, MCScanX (Multiple Collinearity Scan toolkit) was used to analyze intra- and intergenomic synteny [85]. Homologous genes located on the same chromosome closer than 100 kb, and with sequence similarity and sequence coverage > 75%, were regarded as tandem replicated genes [86–88].

### Interaction analysis between GRF and GIF family proteins

We constructed a protein–protein interaction network between all GRF and GIF proteins identified in the same species using the online software STRING (http://string-db.org/) [35]. Selected organisms were same to the species analyzed; the confidence level of minimum required interaction score parameters was set to 0.3, and other parameters were set to the default values [89]. The results were visualized using Cytoscape 3.8.2 [90].

### Split-ubiquitin yeast two-hybrid (Y2H) assay

Split-ubiquitin yeast two-hybrid system was used to examine interactions between the proteins of GRF and GIF. Firstly, the CDS of *GRF* and *GIF* genes were cloned into the pDHB1 and pPR3-N vectors, respectively. Then, the vectors were transformed into yeast strain NMY51 and plated on SD/−Leu/−Trp (SD-LT). Finally, the interactions between the proteins of GRFs and GIFs were assessed by growth of the yeast colonies on SD/−Leu/−Trp/−His (SD LTH) and SD/−Leu/−Trp/−Hiss/−Ade/ (SD LTHA). Yeast cells transformed with pDHB1-largeT and pDSL-P53 vectors were used as positive interaction controls.

### Expression analysis of GRF and GIF family genes

The expression datasets of *A. thaliana* (accession number: GSE680), *P. trichocarpa* (accession number: GSE13990), and *Z. mays* (accession number: GSE27004) were obtained from the Gene Expression Omnibus (GEO, https://www.ncbi.nlm.nih.gov/geo/). The *P. patens* expression dataset was acquired from The Bio-Analytic Resource for Plant Biology (http://bar.utoronto.ca/). The RNA-Seq raw data of *S. tamariscina* (PRJNA507602), *G. biloba* (PRJNA473396), and *O. sativa* (PRJNA243371) were downloaded from the NCBI Sequence Read Archive (SRA, https://www.ncbi.nlm.nih.gov/sra/). The FASTQ data converted from SRA data were subjected to quality control to remove adaptors and filter low quality reads using Fastp software [91], and subsequently compared with the respective reference genomes to obtain the gene expression dataset. Additionally, the *M. polymorpha* expression dataset was retrieved from a previous study [92]. The expression datasets of *GRF* and *GIF* genes were log_2_ transformed, and heatmaps were generated using TBtools [93].

### Supplementary Information

**Additional file 1.**

## Data Availability

The datasets generated and/or analyzed during the current study are available in the NCBI Gene Expression Omnibus database with accession number GSE680, GSE13990 and GSE27004 as well as SRA database with accession number PRJNA507602, PRJNA473396 and PRJNA243371.
