# Supplementary material for: Genome-wide molecular evolution analysis of the GRF and GIF gene families in Plantae (Archaeplastida)
Source: BMC Genomics. 2024 Jan 18;25:74. doi: 10.1186/s12864-024-10006-w (PMC10795294; doi:10.1186/s12864-024-10006-w)
Supplement: Supplementary file 1 — Additional file 1. [file 12864_2024_10006_MOESM1_ESM.zip › Supplementary Files/Figure S1.pdf]

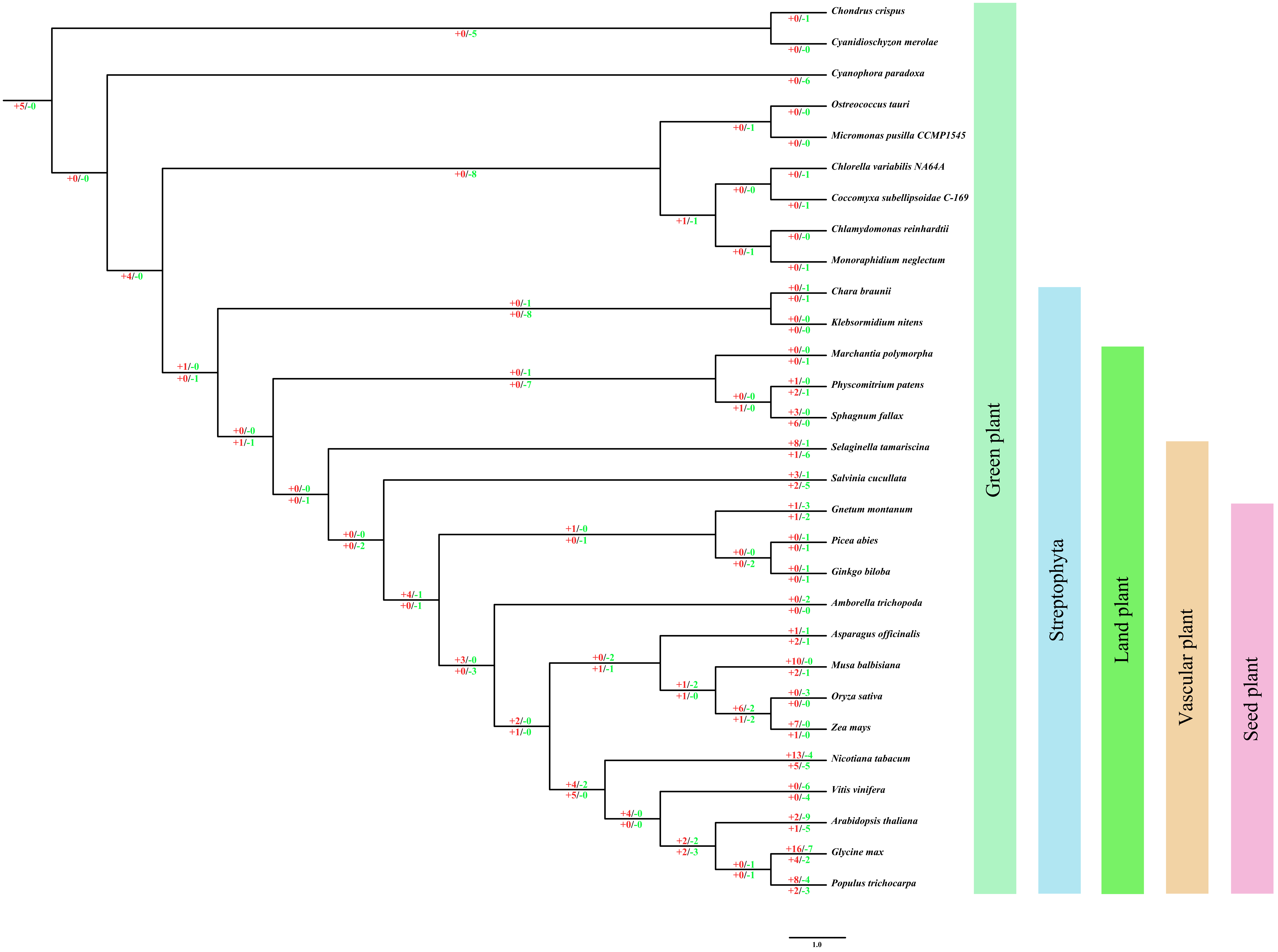

Figure S1. Gene gain and loss of GRF and GIF in the evolution of 29 species. Numbers above and below each branch indicate the numbers of gains (+) and losses (–) of *GRF* and *GIF* genes, respectively.
