# Supplementary material for: Genome-wide molecular evolution analysis of the GRF and GIF gene families in Plantae (Archaeplastida)
Source: BMC Genomics. 2024 Jan 18;25:74. doi: 10.1186/s12864-024-10006-w (PMC10795294; doi:10.1186/s12864-024-10006-w)
Supplement: Supplementary file 1 — Additional file 1. [file 12864_2024_10006_MOESM1_ESM.zip › Supplementary Files/Figure S3.pdf]

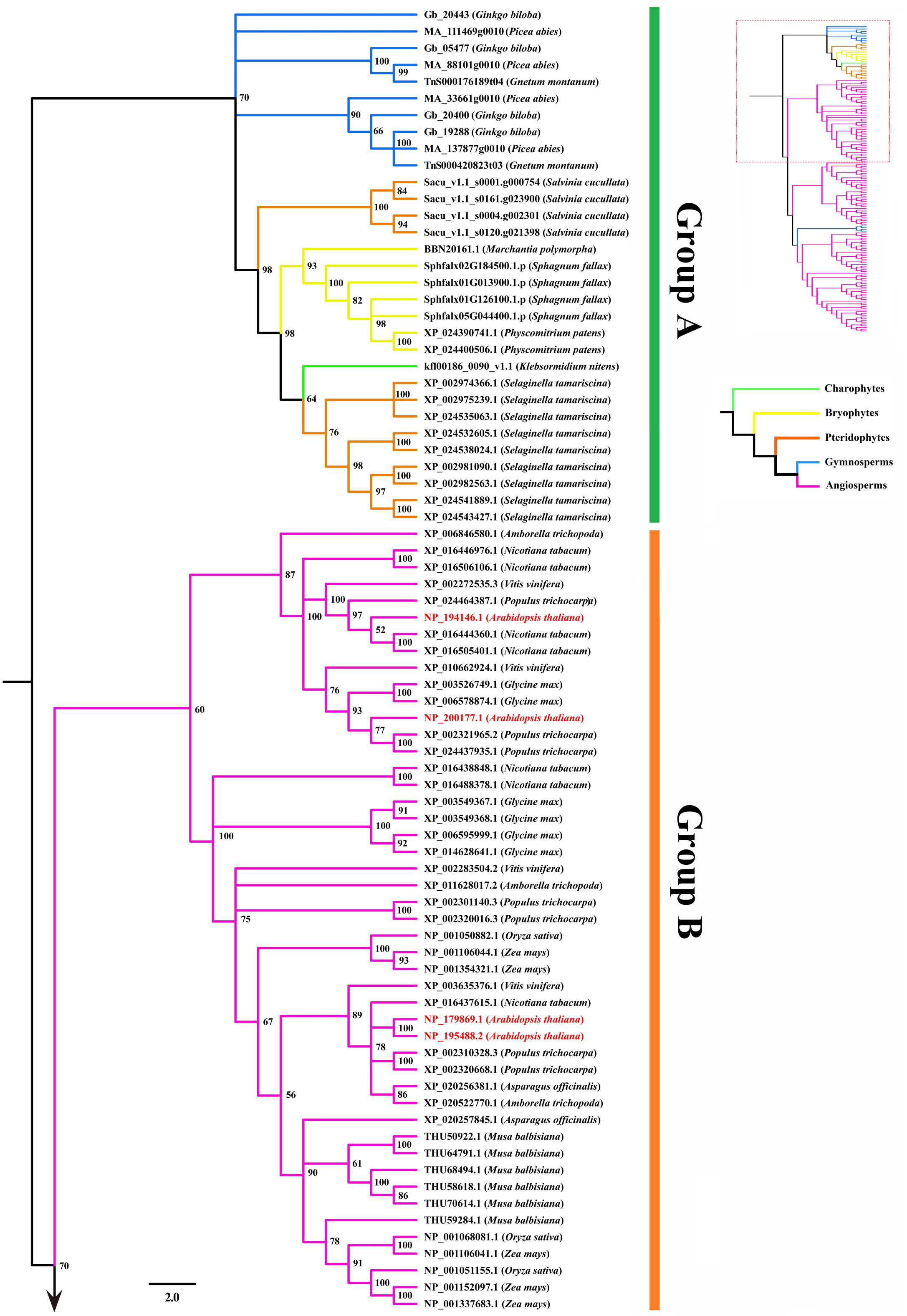

Gb_20443 (Ginkgo biloba)
MA_111469g0010 (Picea abies)
Gb_05477 (Ginkgo biloba)
MA_88101g0010 (Picea abies)
TnS000176189t04 (Gnetum montanum)
MA_33661g0010 (Picea abies)
Gb_20400 (Ginkgo biloba)
Gb_19288 (Ginkgo biloba)
MA_137877g0010 (Picea abies)
TnS000420823t03 (Gnetum montanum)
Sacu_v1.1_s0001.g000754 (Salvinia cucullata)
Sacu_v1.1_s0161.g023900 (Salvinia cucullata)
Sacu_v1.1_s0004.g002301 (Salvinia cucullata)
Sacu_v1.1_s0120.g021398 (Salvinia cucullata)
BBN20161.1 (Marchantia polymorpha)
Sphfalx02G184500.1.p (Sphagnum fallax)
Sphfalx01G013900.1.p (Sphagnum fallax)
Sphfalx01G126100.1.p (Sphagnum fallax)
Sphfalx05G044400.1.p (Sphagnum fallax)
XP_024390741.1 (Physcomitrium patens)
XP_024400506.1 (Physcomitrium patens)
kfl00186_0090_v1.1 (Klebsormidium nitens)
XP_002974366.1 (Selaginella tamariscina)
XP_002975239.1 (Selaginella tamariscina)
XP_024535063.1 (Selaginella tamariscina)
XP_024532605.1 (Selaginella tamariscina)
XP_024538024.1 (Selaginella tamariscina)
XP_002981090.1 (Selaginella tamariscina)
XP_002982563.1 (Selaginella tamariscina)
XP_024541889.1 (Selaginella tamariscina)
XP_024543427.1 (Selaginella tamariscina)
XP_006846580.1 (Amborella trichopoda)
XP_016446976.1 (Nicotiana tabacum)
XP_016506106.1 (Nicotiana tabacum)
XP_002272535.3 (Vitis vinifera)
XP_024464387.1 (Populus trichocarpa)
NP_194146.1 (Arabidopsis thaliana)
XP_016444360.1 (Nicotiana tabacum)
XP_016505401.1 (Nicotiana tabacum)
XP_010662924.1 (Vitis vinifera)
XP_003526749.1 (Glycine max)
XP_006578874.1 (Glycine max)
NP_200177.1 (Arabidopsis thaliana)
XP_002321965.2 (Populus trichocarpa)
XP_024437935.1 (Populus trichocarpa)
XP_016438848.1 (Nicotiana tabacum)
XP_016488378.1 (Nicotiana tabacum)
XP_003549367.1 (Glycine max)
XP_003549368.1 (Glycine max)
XP_006595999.1 (Glycine max)
XP_014628641.1 (Glycine max)
XP_002283504.2 (Vitis vinifera)
XP_011628017.2 (Amborella trichopoda)
XP_002301140.3 (Populus trichocarpa)
XP_002320016.3 (Populus trichocarpa)
NP_001050882.1 (Oryza sativa)
NP_001106044.1 (Zea mays)
NP_001354321.1 (Zea mays)
XP_003635376.1 (Vitis vinifera)
XP_016437615.1 (Nicotiana tabacum)
NP_179869.1 (Arabidopsis thaliana)
NP_195488.2 (Arabidopsis thaliana)
XP_002310328.3 (Populus trichocarpa)
XP_002320668.1 (Populus trichocarpa)
XP_020256381.1 (Asparagus officinalis)
XP_020522770.1 (Amborella trichopoda)
XP_020257845.1 (Asparagus officinalis)
THU50922.1 (Musa balbisiana)
THU64791.1 (Musa balbisiana)
THU68494.1 (Musa balbisiana)
THU58618.1 (Musa balbisiana)
THU70614.1 (Musa balbisiana)
THU59284.1 (Musa balbisiana)
NP_001068081.1 (Oryza sativa)
NP_001106041.1 (Zea mays)
NP_001051155.1 (Oryza sativa)
NP_001152097.1 (Zea mays)
NP_001337683.1 (Zea mays)
Group A
Group B
Charophytes
Bryophytes
Pteridophytes
Gymnosperms
Angiosperms
2.0

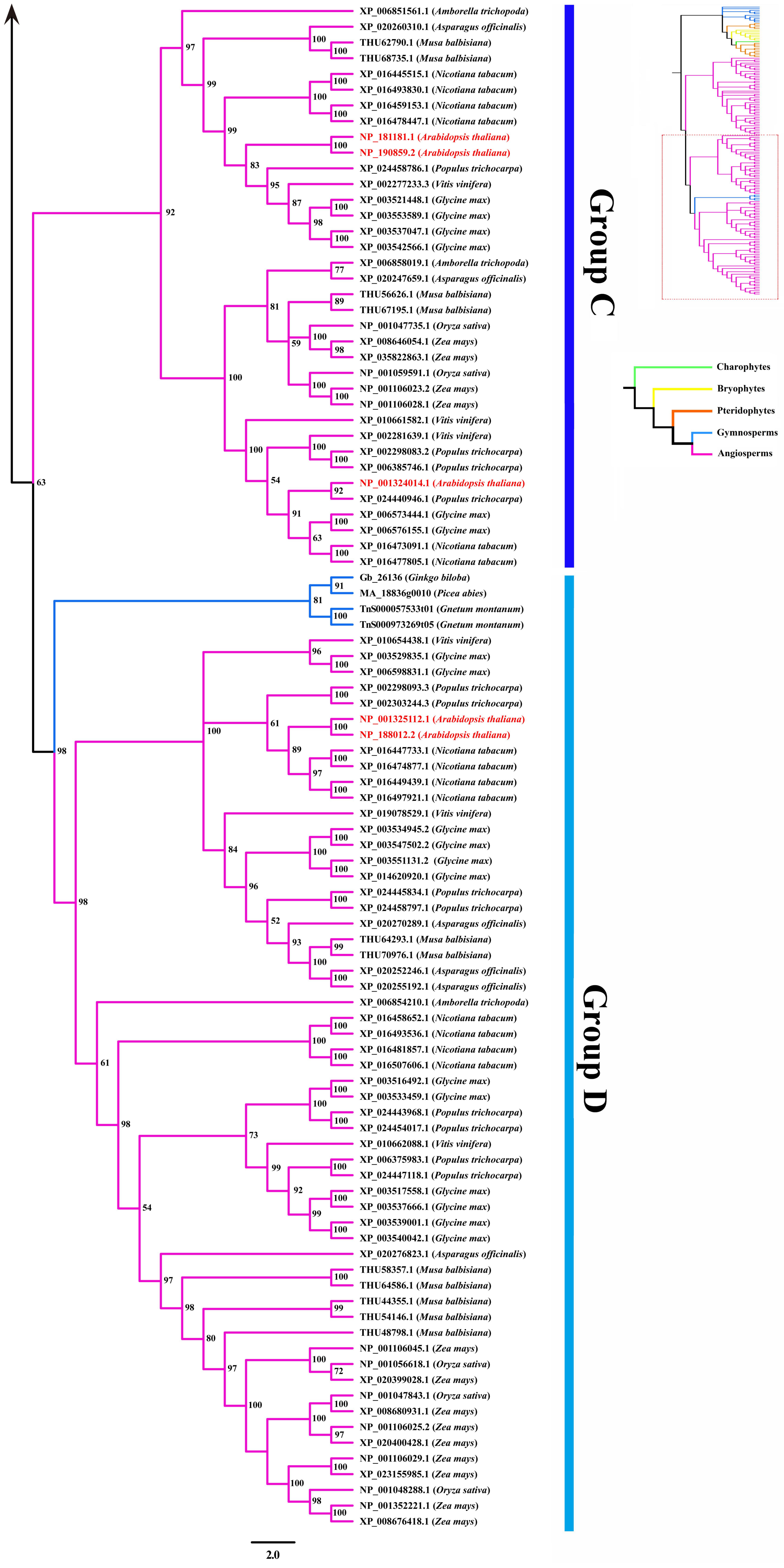

Figure S3. Phylogenetic tree of the *GRF* gene family constructed by the Bayesian method. *GRF* genes from *A. thaliana* are shown in red.
