# Supplementary figures and images for: Genome-wide molecular evolution analysis of the GRF and GIF gene families in Plantae (Archaeplastida)
Source: BMC Genomics. 2024 Jan 18;25:74. doi: 10.1186/s12864-024-10006-w (PMC10795294; doi:10.1186/s12864-024-10006-w)

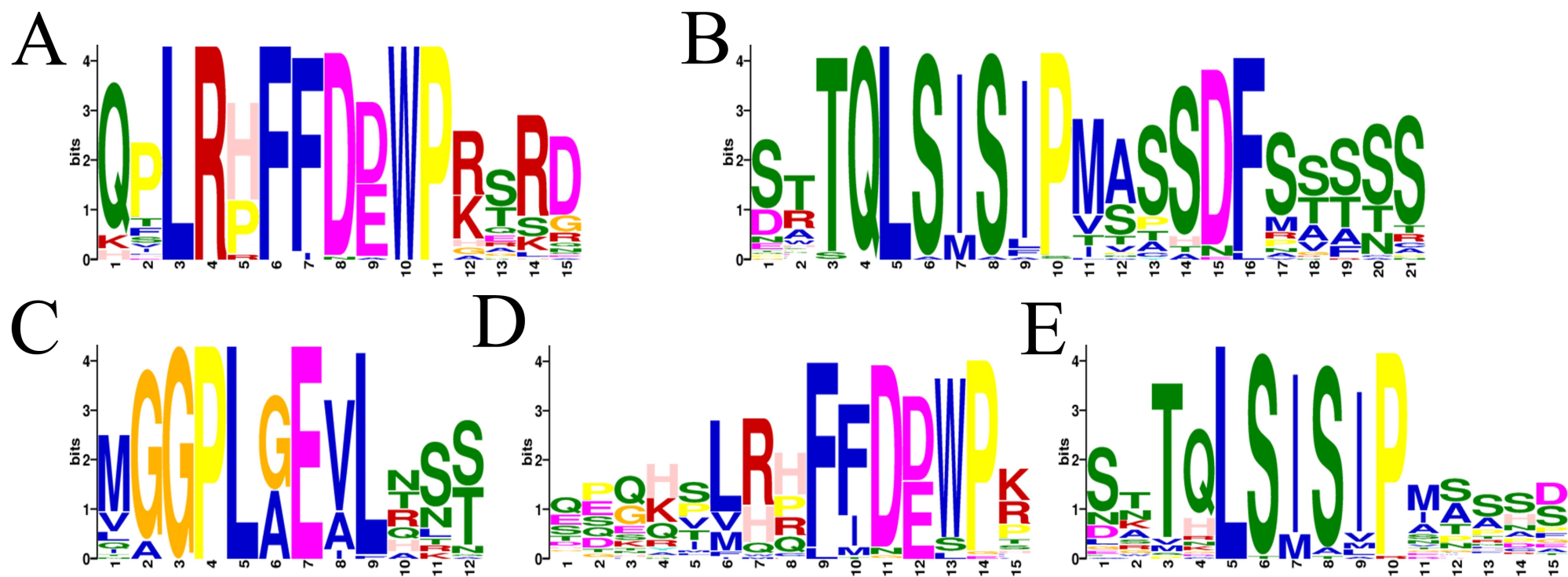

Figure S5. Analysis of amino acid motifs in the C-termini of GRF family proteins.

Supplement: Supplementary file 1 — Additional file 1. [file 12864_2024_10006_MOESM1_ESM.zip › Supplementary Files/Figure S6.pdf]

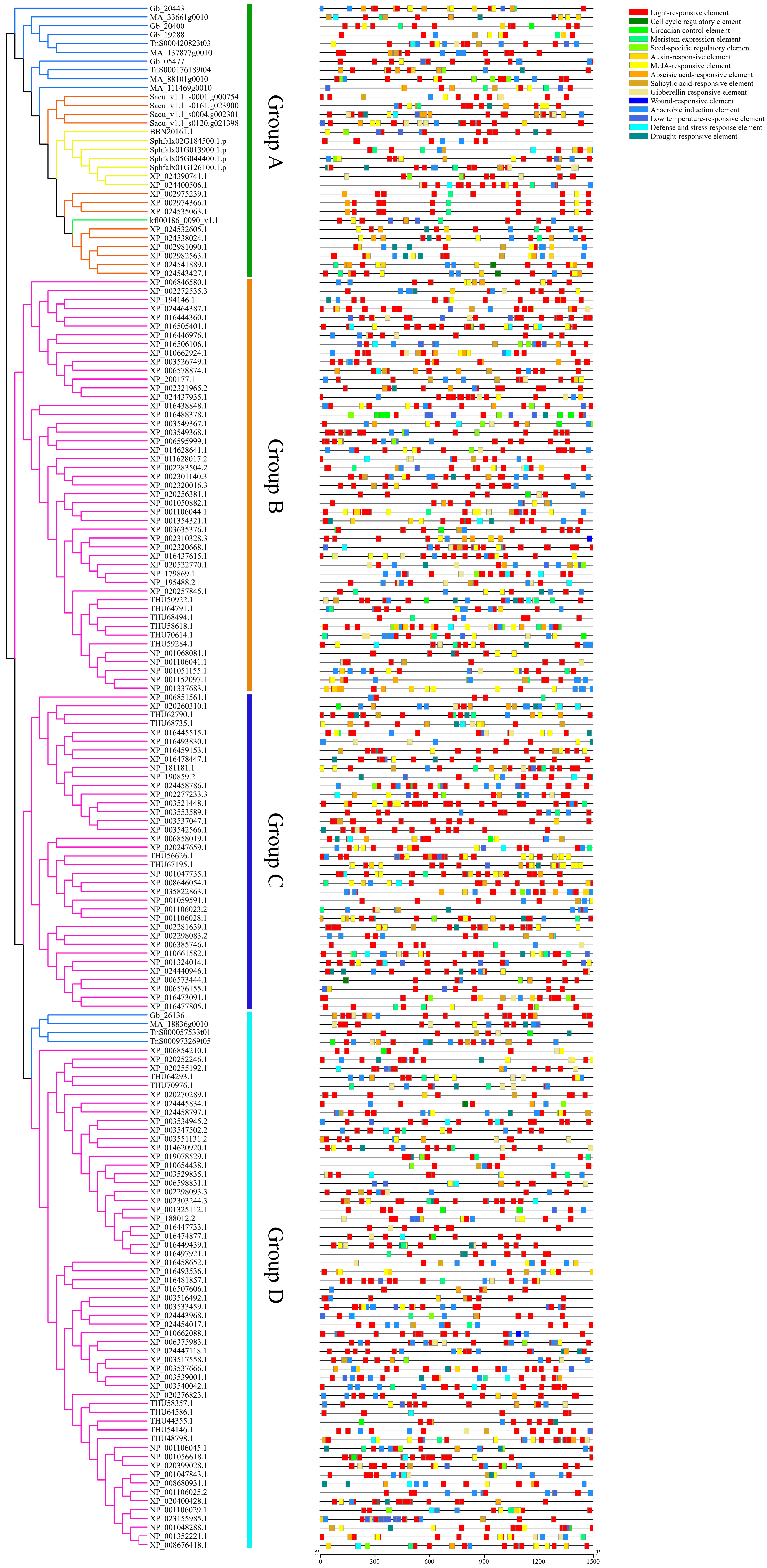

Figure S7. Analysis of cis-acting elements in the promoter regions of *GRF* family genes.

Supplement: Supplementary file 1 — Additional file 1. [file 12864_2024_10006_MOESM1_ESM.zip › Supplementary Files/Figure S7.pdf]

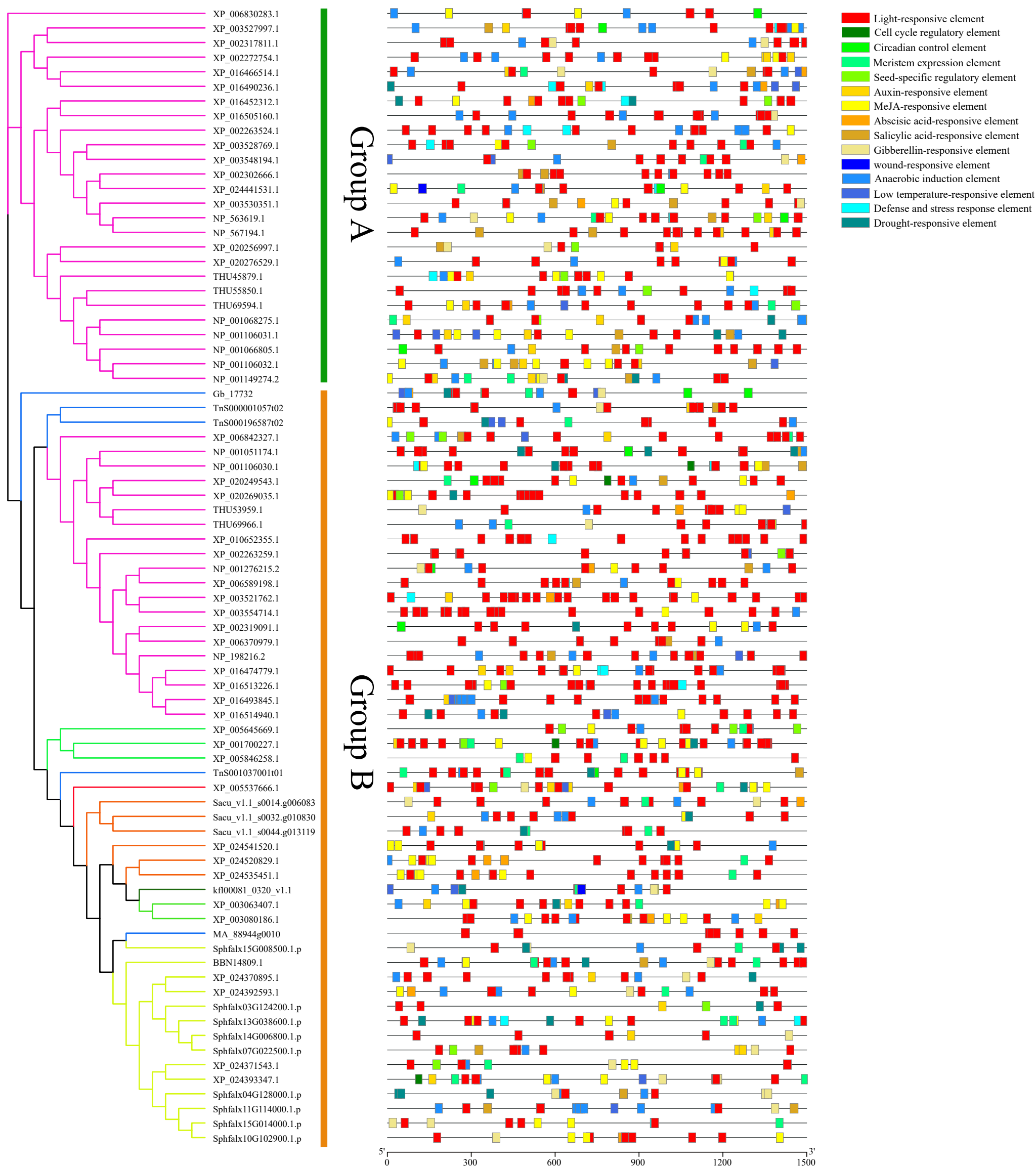

Figure S8. Analysis of cis-acting elements in the promoter regions of *GIF* family genes.

Supplement: Supplementary file 1 — Additional file 1. [file 12864_2024_10006_MOESM1_ESM.zip › Supplementary Files/Figure S8.pdf]
